# The metastasis suppressor CD82/KAI1 inhibits fibronectin adhesion-induced epithelial-to-mesenchymal transition in prostate cancer cells by repressing the associated integrin signaling

**DOI:** 10.18632/oncotarget.13767

**Published:** 2016-12-01

**Authors:** Jaeseob Lee, Hee-Jung Byun, Moon-Sung Lee, Young-June Jin, Dooil Jeoung, Young-Myeong Kim, Hansoo Lee

**Affiliations:** ^1^ Department of Biological Sciences, College of Natural Sciences, Kangwon National University, Chunchon, Kangwon-do 200-701, Republic of Korea; ^2^ BIT Medical Convergence Graduate Program, Kangwon National University, Chunchon, Kangwon-do 200-701, Republic of Korea; ^3^ Department of Biochemistry, College of Natural Sciences, Kangwon National University, Chunchon, Kangwon-do 200-701, Republic of Korea; ^4^ Department of Molecular and Cellular Biochemistry, School of Medicine, Kangwon National University, Chunchon, Kangwon-do 200-701, Republic of Korea

**Keywords:** CD82, EMT, fibronectin matrix, integrin signaling, cancer invasion

## Abstract

The transmembrane protein CD82/KAI1 suppresses the metastatic potential of various cancer cell types. Moreover, decrease or loss of CD82 expression is closely associated with malignancy and poor prognosis in many human cancers including prostate cancer. Despite intense scrutiny, the mechanisms underlying the metastasis-suppressing role of CD82 are still not fully understood. Here, we found that a fibronectin matrix induced mesenchymal phenotypes in human prostate cancer cells with no or low CD82 expression levels. However, high CD82 expression rendered prostate cancer cells to have intensified epithelial characteristics upon fibronectin engagement, along with decreased cell motility and invasiveness. The CD82 function of inhibiting fibronectin-induced epithelial-to-mesenchymal transition (EMT) was dependent not only on CD82 interactions with fibronectin-binding α_3_β_1_/α_5_β_1_ integrins but also on the integrin-mediated intracellular signaling events. Notably, CD82 attenuated the FAK-Src and ILK pathways downstream of the fibronectin-receptor integrins. Immunofluorescence staining of human prostate cancer tissue specimens illustrated a negative association of CD82 with EMT-related gene expression as well as prostate malignancy. Altogether, these results suggest that CD82 suppresses EMT in prostate cancer cells adhered to the fibronectin matrix by repressing adhesion signaling through lateral interactions with the associated α_3_β_1_ and α_5_β_1_ integrins, leading to reduced cell migration and invasive capacities.

## INTRODUCTION

The membrane protein CD82 is a member of the tetraspanin superfamily that contains four transmembrane domains, short N- and C-terminal cytoplasmic domains, two extracellular loops, and a small intracellular loop [1, 2]. Tetraspanins are implicated not only in a broad range of physiological processes, but also in pathological situations such as cancer invasion and metastasis [2, 3]. Tetraspanins form large multi-molecular complexes that are clustered in specialized membrane domains known as tetraspanin-enriched microdomains (TEMs) [1, 4]. In TEMs, tetraspanin proteins are not only physically complexed with other membrane proteins including integrins, growth factor receptors, and proteases, but also associate with signaling molecules such as PKC, PI4-kinase, and small GTPases [1, 4–7]. Since tetraspanin proteins have no enzymatic activity, tetraspanins seems instead to function by regulating the activity, function, or membrane trafficking of the associated molecules in the TEMs through lateral interactions and crosstalk.

KAI1, which is identical to CD82, was initially identified as a metastasis suppressor of prostate cancer [8]. Enforced expression of CD82/KAI1 by gene transfection significantly reduced lung metastases of rat prostate cancer cells, without affecting primary tumor growth. In subsequent studies, the metastasis suppressor function of CD82/KAI1 was demonstrated in many other cancer cell types including breast cancer, melanoma, sarcoma, pancreatic cancer, and hepatocarcinoma cells [9–13]. The importance of CD82/KAI1 in malignant cancer progression has been supported by an inverse correlation between its expression levels and the metastases of a variety of human cancers. Despite wide investigations, many aspects of how CD82/KAI1 specifically suppresses cancer metastasis are not yet fully determined.

Key steps in malignant cancer progression are represented by the changes associated with epithelial-to-mesenchymal transition (EMT) [14, 15]. During EMT, polarized epithelial cells downregulate E-cadherin, which results in the dissolving of cell-cell adhesion, the disruption of cell polarity, and the upregulation of multiple mesenchymal-associated proteins to become motile mesenchymal cells [14, 16, 17]. Cancer cells undergoing EMT acquire invasive behavior and consequently metastatic competence, along with increased resistance to cell death and stemness [14, 15, 18]. The EMT process in both normal and transformed cells can be initiated by many signaling pathways, including those triggered by TGF-β1, EGF, HGF, FGF, Wnts, Notch, and many other extracellular factors [18]. Adhesion signaling initiated by integrin-matrix interactions also contributes to EMT in various cell types. Several integrins including α_2_β_1_, α_3_β_1_, α_5_β_1_, and α_v_β_3_ subtypes were shown to be involved in the EMT program [19–24]. Integrin downstream signaling mediators such as focal adhesion kinase (FAK) and integrin-linked kinase (ILK) also play a role in inducing EMT [25–29]. Additionally, increased deposition of fibronectin into the matrix during EMT further accelerates the EMT process through continual stimulation of integrin signaling [30]. Previously, we have found that CD82 not only suppresses fibronectin expression but also the activation of β_1_ integrins and their downstream signaling in prostate cancer cells [31]. Therefore, we have postulated that the CD82 function of repressing integrin-matrix interactions and subsequent intracellular signaling could be linked to the CD82 effects on matrix-dependent EMT.

In the present study, using CD82 loss- and gain-of function approaches on three human prostate cancer cell lines with invasive and metastatic potential, we identified CD82 as a potent inhibitor of matrix adhesion-dependent EMT in prostate cancer cells, providing a clue for understanding how CD82 suppresses the tumor cell-intrinsic invasive and metastatic potential. This study shows that high CD82 expression blocks fibronectin-induced EMT through repression of adhesion signaling mediated by CD82-interacting fibronectin-receptor integrins, which not only suggests a mechanism of EMT inhibition by CD82, but also supports the importance of integrin signaling in EMT.

## RESULTS

### High CD82 expression blocks development of mesenchymal phenotypes in human prostate cancer cells adhered to fibronectin

**Figure 1:**
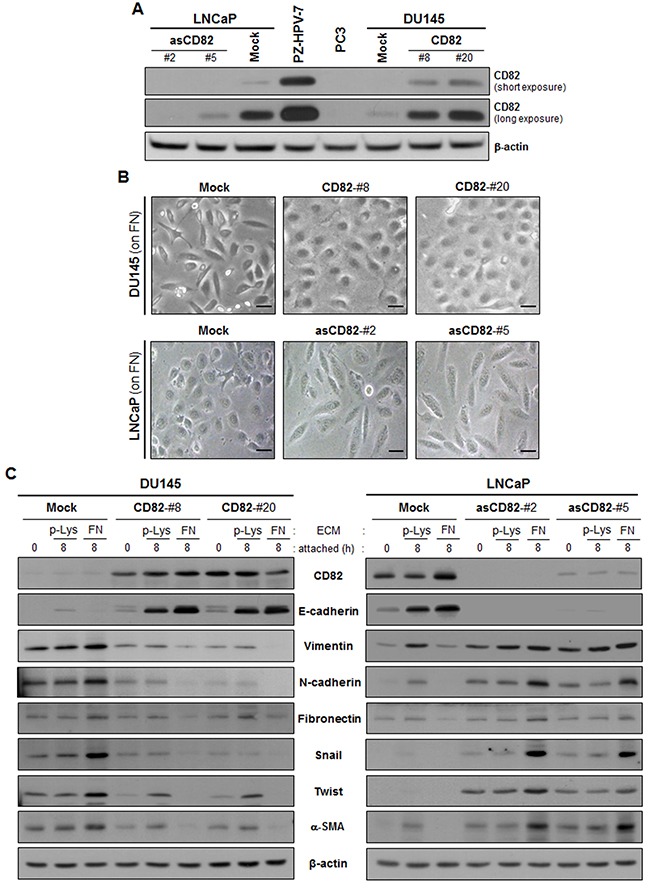
CD82 inhibits fibronectin adhesion-induced EMT in prostate cancer cells **A.** Human prostate epithelial (PZ-HPV-7) and cancer (DU145, LNCaP, and PC3) cell lines and stably CD82-transfected DU145 and antisense CD82 fragment-transfected LNCaP cell clones were assessed for CD82 expression levels through immunoblotting analysis. **B.** Stably CD82-transfected DU145 and antisense CD82 fragment-transfected LNCaP cell clones grown on fibronectin (FN) were viewed under a phase-contrast microscope. Scale bar, 10 μm. **C.** The cells were seeded onto plates precoated with poly-L(+)-lysine (p-Lys) or FN and cultured for the indicated time periods. Expression of E-cadherin and mesenchymal marker proteins was examined through immunoblotting analysis using antibodies specific to each protein.

We first examined CD82 expression levels in three prostate cancer cell lines derived from metastatic sites in prostate cancer patients, LNCaP from a lymph node metastasis, DU145 from a bone metastasis, and PC3 from a brain metastasis. Although CD82 was expressed in LNCaP and DU145 cells, CD82 protein levels in these two prostate cancer cell lines were significantly lower than those in a human prostate epithelial cell line, PZ-HPV-7 (Figure 1A). Furthermore, PC3 cells were found to be deficient in CD82 protein, indicating that CD82 was downregulated in all three human prostate cancer cell lines examined. Next, we generated stable CD82 transfectant clones of DU145 cells, which express more CD82 than the parental cell line. We also knockdowned CD82 in LNCaP cells by stable transfection with antisense CD82 cDNA fragment.

Since CD82 associates with fibronectin-binding integrins physically and functionally, we cultured the prostate cancer cells on fibronectin-coated plates and examined cell morphology. Low CD82-expressing DU145 cells showed a spindle-shaped fibroblast-like morphology, while the high CD82-expressing cells displayed a round cobblestone-like shape (Figure 1B). LNCaP cells with high CD82 levels also showed a round epithelial morphology, but CD82 knockdown resulted in a morphological change of LNCaP cells into a fibroblast-like shape. Thus, high CD82 expression inhibits prostate cancer cells adhered to the fibronectin matrix from developing a fibroblast-like mesenchymal morphology. Next, we compared the expression levels of EMT-associated genes between the low and high CD82-expressing cells. As shown in Figure 1C, E-cadherin levels in the low CD82-expressing cells were much lower than those in the high CD82-expressing cells. Moreover, the low CD82-expressing cells contained more mesenchymal proteins such as vimentin, N-cadherin, fibronectin, Snail, Twist, and α-smooth muscle actin (α-SMA) than the high CD82-expressing cells. Notably, in the low CD82-expressing cells, fibronectin significantly increased the abundance of mesenchymal proteins as compared with poly-L(+)-lysine, a cationic polyelectrolyte that minimally activates any type of integrins [32, 33]. In contrast, fibronectin engagement in the high CD82-expressing cells further downregulated mesenchymal proteins and upregulated E-cadherin. A similar result was also observed in PC3 cells devoid of endogenous CD82, where fibronectin upregulated mesenchymal proteins, along with the acquisition of mesenchymal morphology (Supplementary Figure S1). On the contrary, in PC3 cells with ectopically overexpressed CD82 by adenoviral CD82 transduction, fibronectin upregulated E-cadherin instead of mesenchymal proteins, concomitantly with increased cell-cell adhesion. Therefore, it is likely that fibronectin-integrin interactions promote development of mesenchymal phenotypes in prostate cancer cells with no or low CD82 levels, but reinforce an epithelial phenotype in cells with high CD82 levels, implicating differential effects of fibronectin adhesion on the epithelial/mesenchymal state of prostate cancer cells depending on CD82 expression levels. Taken together, the present data demonstrate a CD82 function of the suppression of fibronectin-induced EMT in prostate cancer cells, which may account for the invasion-suppressing role of CD82.

### CD82 decreases the motility and invasiveness of human prostate cancer cells

**Figure 2:**
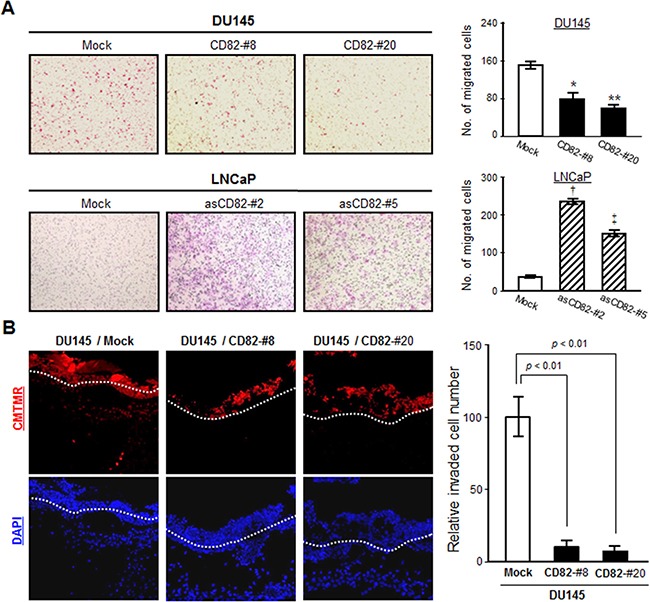
CD82 suppresses chemotactic migration and invasiveness of prostate cancer cells **A.** Chemotactic cell migration assay using Transwell-chamber inserts was performed as described in “Materials and Methods”. Results are the mean ± s.d. from three separate experiments performed in triplicate (*, **, and ‡, *p* < 0.03; †, *p* < 0.01 *versus* mock; Student's *t*-test). **B.** Cells were labeled with a fluorescent cell tracker, CMTMR (red), and seeded atop the chick chorioallantoic membrane (CAM) of 11-day-old chick embryos. After three days incubation, the embryos were frozen and cross-sectioned. CAM sections stained with DAPI (blue) were photographed under a fluorescent microscope. The CAM surface is marked with a dashed line. Cells showing both red and blue fluorescence beneath the CAM surface were counted and expressed as the mean ± s.d. of five sections in each of the four embryos.

Since cells that underwent EMT became more motile and invasive, we compared the migrating abilities between the low and high CD82-expressing cells. In a Transwell-chamber cell migration assay using the conditioned medium from fibroblast cultures as a chemoattractant, the high CD82-expressing cells exhibited a decreased chemotactic movement as compared with the low CD82-expressing cells (Figure 2A). A wound-closure cell migration assay also revealed that CD82 inhibits the chemostatic motility of prostate cancer cells (Supplementary Figure S2A). However, cell proliferation was not affected by CD82 (data not shown). Therefore, CD82 is likely to have a negative motogenic function without any mitogenic effect on prostate cancer cells. When cancer cell invasion was examined using matrigel as a basement membrane barrier, the high CD82-expressing cells displayed decreased invasiveness as compared with the low CD82-expressing cells (Supplementary Figure S2B). An *in vivo* invasion assay using chick embryos also illustrated that high CD82 expression significantly suppressed the invasive capacities of prostate cancer cells (Figure 2B). Overall, these results demonstrate a CD82 function in the suppression of the tumor cell-intrinsic migrating and invasive potential, which corresponds to its EMT-suppressing role.

### CD82 regulation of E-cadherin and Snail expression involves CD82-interacting α_3_β_1_ and α_5_β_1_ integrins

**Figure 3:**
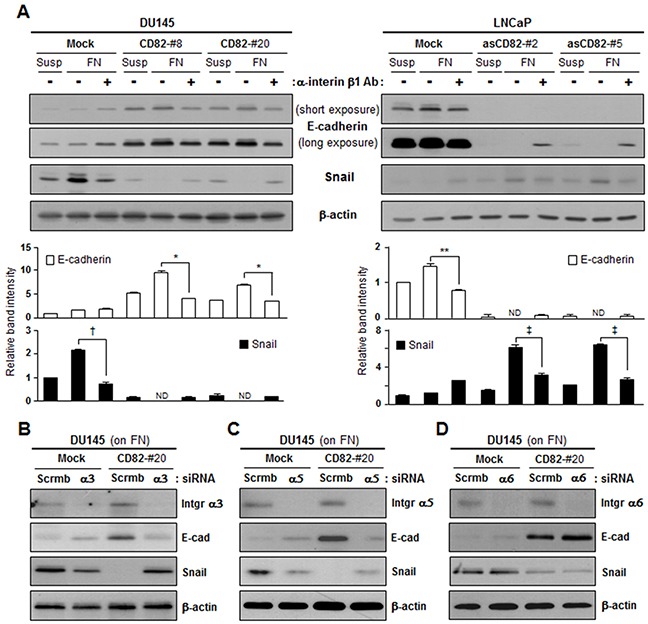
CD82 inhibition of fibronectin-induced EMT is dependent on α_3_β_1_ and α_5_β_1_ integrins **A.** Stably CD82-transfected DU145 and antisense CD82 fragment-transfected LNCaP cell clones suspended in culture medium were pretreated with a mouse normal IgG_1_ or integrin β_1_-blocking IgG_1_ for 2 hours and cultured on plates precoated with fibronectin (FN) for 8 hours. Cells in suspension (Susp), and cells grown onto the plates were examined for expression of E-cadherin and Snail using immunoblotting analysis. Bar graphs under the immunoblots indicate the relative band intensity of E-cadherin and Snail normalized to the loading control (actin). Values are the mean ± s.d. from three separate experiments performed in triplicate (*, **, †, and ‡, *p* < 0.03). ND, not detectable. **B-D.** Cells grown on FN were transfected with either scrambled (scrmb) siRNAs or integrin α_3_ (B), α_5_ (C), or α_6_ (D) subunit-specific siRNAs and then examined for E-cadherin and Snail expression.

To verify involvement of fibronectin-binding integrins in CD82 inhibition of fibronectin adhesion-dependent EMT, we treated the cells with an anti-β_1_ integrin antibody. The antibody blocking of β_1_ integrins prior to fibronectin-integrin interactions attenuated the fibronectin effects on E-cadherin and Snail expression in both the low and high CD82-expressing cells, indicating that fibronectin regulates EMT through β_1_ subunit-containing integrins (Figure 3A). To further identify the subtype of β_1_ integrins, we knockdowned several integrin α subunits including α_3_, α_5_, and α_6_ through siRNA transfection and examined E-cadherin and Snail expression in the cells adhered to fibronectin. In integrin subunit α_3_- and α_5_-null cells that overexpressed CD82, E-cadherin upregulation and Snail downregulation were not observed. However, E-cadherin expression levels were increased and Snail levels were decreased in the CD82-overexpressing cells where α_6_ subunit was knockdowned (Figure 3B-3D). Therefore, CD82 is likely to be involved in the regulation of epithelial and mesenchymal protein expression by working with α_3_β_1_ and α_5_β_1_ integrins functioning as fibronectin-receptors.

**Figure 4:**
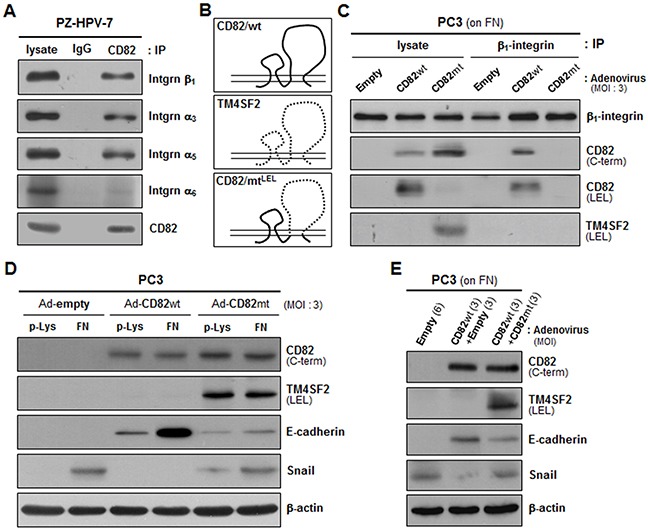
Intramembrane interactions of CD82 with β_1_ integrins are essential for CD82 inhibition of fibronectin-induced EMT **A.** PZ-HPV-7 prostate epithelial cells were lysed with Brij 97 detergent, and immunoprecipitation (IP) was performed with normal mouse IgG or anti-CD82 antibody. The immunoprecipitates were analyzed by immnublotting using anti-integrin β_1_, α_3_, α_5_, or α_6_ antibody. **B.** CD82 mutant cDNA, which encodes CD82 with a large extracellular loop (LEL) substituted with that of TM4SF2 as illustrated, was generated by PCR and subcloned into the pAdEasy-1 adenoviral vector to produce recombinant adenovirus. **C.** CD82-deficient PC3 prostate cancer cells grown on fibronectin (FN) were infected with adenovirus containing a wild-type (wt) or mutant (mt) CD82 expression construct, and Brij 97 detergent lysates were subjected to immunoprecipitation with an anti-β_1_ integrin antibody followed by immunoblotting analysis using antibodies that recognize the C-terminus or LEL of CD82 and the LEL of TM4SF2. **D.** PC3 cells grown on poly-L(+)-lysine (p-Lys) or FN were infected with adenovirus containing a wt- or mt-CD82 expression construct and then assessed for the protein levels of E-cadherin and Snail. **E.** PC3 cells grown on FN were infected with wt-CD82 construct-containing adenovirus either alone or together with mt-CD82 construct-containing adenovirus and examined for E-cadherin and Snail expression. Numbers in parentheses represent the MOI values of adenovirus.

Since CD82 was physically complexed with α_3_β_1_ and α_5_β_1_ integrins in human prostate epithelial cells (Figure 4A), similar to other adherent cells [34, 35], we examined whether intramembrane interactions of CD82 with the fibronectin-receptor integrins are a prerequisite for the CD82 function of upregulating E-cadherin and downregulating Snail. A CD82 mutant in which the large extracellular loop (LEL) region of CD82 was replaced with the corresponding region from another tetraspanin, TM4SF2, was not co-immunoprecipitated with β_1_ integrins (Figure 4B and 4C). Unlike the wild-type CD82 that associates with β_1_ integrins, this LEL mutant of CD82 was not able to downregulate Snail in PC3 cells devoid of endogenous CD82 (Figure 4D). Fibronectin also minimally upregulated E-cadherin in the CD82 LEL mutant-expressing cells as compared to the wild-type CD82-expressing cells. Furthermore, the effects of wild-type CD82 on E-cadherin and Snail expression were attenuated by the CD82 LEL mutant (Figure 4E). Collectively, these results suggest that CD82 influences the expression of EMT-associated genes through its lateral interactions with fibronectin-binding α_3_β_1_ and α_5_β_1_ integrins.

### CD82 inhibits fibronectin-induced EMT by repressing intracellular adhesion signaling cascades downstream of the fibronectin-binding integrins

**Figure 5:**
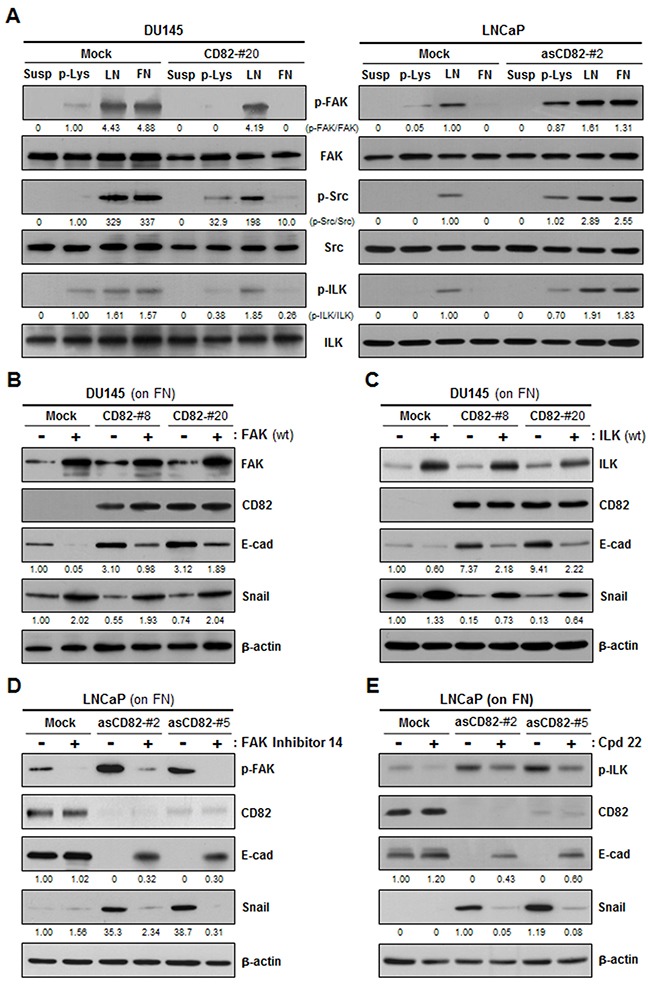
CD82 suppresses fibronectin-induced EMT by attenuating fibronectin-binding integrin signaling cascades including the FAK-Src and ILK pathways **A.** DU145 and LNCaP cell transfectant clones were cultured on plates precoated with poly-L(+)-lysine (p-Lys), laminin (LN), or fibronectin (FN) for 8 hours. Phosphorylation levels of FAK, Src, and ILK in the cells in suspension (Susp) and cells grown onto the plates were assessed by immunoblotting analysis using antibodies recognizing phospho-FAK^(Tyr-925)^, phospho-Src^(Tyr-416)^, or phospho-ILK^(Thr-173)^. Shown are representative immunoblots from three separate experiments performed in triplicate. Numbers under the immunoblots indicate the relative band intensity of the phosphorylated protein normalized to the protein level. **B, C.** DU145 cell transfectant clones grown on FN were transiently transfected with a wild-type FAK (B) or ILK (C) expression construct, and examined for E-cadherin and Snail expression. The protein level was normalized to the loading control (actin) level. **D, E.** LNCaP cell transfectant clones were pretreated with inhibitors specific to FAK (FAK inhibitor 14) (D) or ILK (Cpd22) (E) at a concentration of 0.1 μM for 2 hours. Cells were cultured on FN-coated plates for 8 hours and then assessed for the protein levels of E-cadherin and Snail as well as the phosphorylation levels of FAK (D) and ILK (E).

Integrins activated by interactions with the matrix transduce adhesion signals into the cell through the FAK-Src and ILK pathways. Among DU145 and LNCaP cell transfectant clones, phosphorylation levels of FAK, Src, and ILK in the low CD82-expressing cells were significantly increased by both fibronectin and laminin (Figure 5A). However, in the high CD82-expressing cells, fibronectin did not induce phosphorylation of FAK, Src, and ILK, differently from laminin, suggesting that CD82 specifically suppresses the signaling activity of fibronectin-receptor integrins. To confirm whether CD82 inhibition of fibronectin-mediated EMT is dependent on fibronectin-receptor signaling events, we overexpressed wild-type FAK and ILK in prostate cancer cells through gene transfection. Upon fibronectin engagement, both FAK- and ILK-overexpressing cells displayed decreased E-cadherin and increased Snail expression as compared with the counterpart control transfectant cells (Figure 5B and 5C). Importantly, E-cadherin downregulation and Snail upregulation by FAK and ILK overexpression occurred not only in the low CD82-expressing cells but also in the high CD82-expressing cells, implying that intensified integrin intracellular signaling incapacitates CD82 from suppressing matrix-dependent EMT. Meanwhile, chemical inhibitors specific to FAK and ILK restrained fibronectin from downregulating E-cadherin and upregulating Snail in the low CD82-expressing cells (Figure 5D and 5E), supporting an involvement of the FAK and ILK pathways in fibonectin-induced EMT. However, in the high CD82-expressing cells adhered to fibronectin, E-cadherin and Snail expression were not affected by these FAK and ILK inhibitors. Thus, CD82 played a role similar to these chemical inhibitors to block fibronectin-induced EMT as well as integrin downstream signaling. Taken together, the current data suggest that high CD82 expression blocks fibronectin adhesion-induced EMT in prostate cancer cells through the repression of associated α_3_β_1_/α_5_β_1_ integrin-mediated signaling events.

### CD82 levels in human prostate cancer specimens are closely associated with E-cadherin but inversely correlated with mesenchymal protein levels and malignant states of prostate cancers

**Figure 6:**
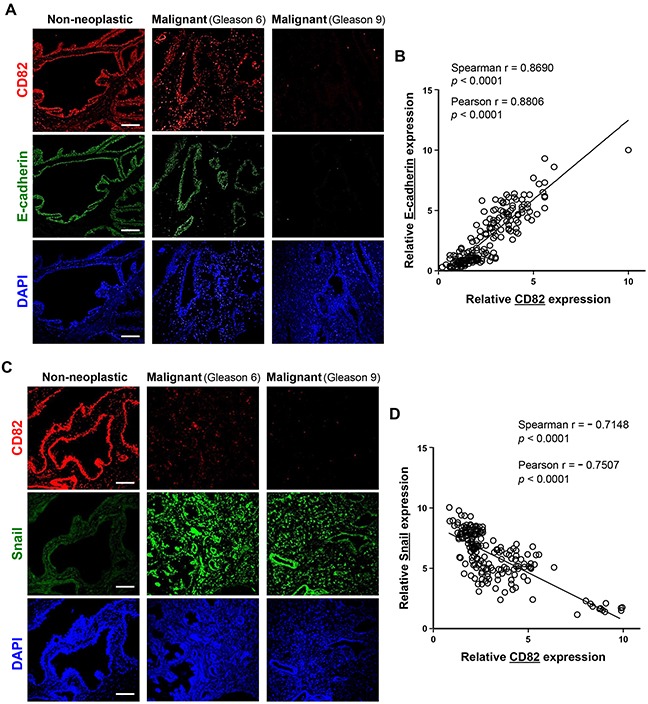
Expression levels of CD82, E-cadherin, and Snail in human prostate cancer tissue samples **A, C.** Two microarray slides, which contain malignant samples in duplicates from 90 prostate cancer patients along with 12 non-neoplastic control human prostate tissue specimens, were subjected to immunofluorescence staining using rabbit anti-CD82 antibody, together with mouse anti-E-cadherin (A) or anti-Snail (C) antibody. Following incubation with fluorescent secondary antibodies, photomicrographs were taken under a fluorescent microscope. Shown are representative fluorescence images of the immunostained tissue sections. Scale bar, 100 μm. **B, D.** Expression levels of CD82, E-cadherin (B), and Snail (D) were assessed by fluorescence intensity of target proteins. (B) Expression levels of CD82 were significantly proportional to those of E-cadherin. (D) An inverse correlation was found between CD82 and Snail levels. Numbers represent the statistical data from Spearman and Pearson correlation coefficient tests.

We examined the expression levels of CD82, E-cadherin, and mesenchymal proteins including Snail, α-smooth muscle actin (α-SMA), and vimentin in human prostate cancer tissues from patients through immunofluorescence staining of tissue microarray sections comprising 90 prostate adenocarcinomas with different malignant stages and 12 non-neoplastic prostate tissue samples. Malignant tissue sections of prostate cancer displayed significantly decreased expression levels of CD82 and E-cadherin as compared with the corresponding non-neoplastic sections (Figure 6A, Table 1). In contrast, Snail levels in the malignant tissues were much higher than those in normal prostate tissues (Figure 6C). Importantly, CD82 expression was positively correlated with E-cadherin expression (Figure 6B). However, an inverse correlation was found between CD82 and Snail levels (Figure 6D). Similar to Snail, both α-SMA and vimentin not only exhibited elevated expression levels in malignant tissues as compared to normal prostate tissues, but also showed negative relationships with CD82 expression (Supplementary Figure S3). Among the malignant tissues, the higher the Gleason grade, the less CD82- and E-cadherin-positive cells were found (Table 1), implicating a negative association of CD82 and E-cadherin with malignant progression of human prostate. On the contrary, mesenchymal proteins such as Snail, α-SMA, and vimentin displayed positive relationships with malignant states of prostate cancers (Table 1, Supplementary Table S1). Taken together, these findings suggest that CD82 is co-downregulated with E-cadherin during malignant prostate cancer development, which provokes prostate tumor cells to undergo EMT.

**Table 1:** Expression of CD82, E-cadherin, and Snail in human prostate cancer tissue samples

Relative expression of target protein^*a*^	Percentage of cases of histologic type (n, total number)*^*b*^*			
	Non-neoplasitic (n=12)	Malignant		
		≤ Gleason 7 (n=66)	Gleason 8 ≤ (n=101)	*p* value^*c*^
**CD82**				
1	0% (0)	31.8% (21)	61.4% (62)	< 0.0001
2	25.0% (3)	59.1% (39)	34.7% (35)	
3	66.7% (8)	9.1% (6)	4.0% (4)	
4	8.3% (1)	0% (0)	0% (0)	
**E-cadherin**				
1	0% (0)	39.4% (26)	55.4% (56)	< 0.0001
2	25.0% (3)	33.3% (22)	31.7% (32)	
3	50.0% (6)	25.8% (17)	12.9% (13)	
4	25.0% (3)	1.5% (1)	0% (0)	
**Snail**				
1	100% (12)	3% (2)	0% (0)	< 0.0001
2	0% (0)	36.4% (24)	2% (2)	
3	0% (0)	60.6% (40)	38.6% (39)	
4	0% (0)	0% (0)	59.4% (60)	

a Staining intensity relative to the highest intensity was scored as follows: 1, < 25%; 2, 25~50%; 3, 50~75%; 4, >75%

b Proportion of tumors with respective score out of total tissue number (*n*) examined is given.

c Statistical significance was determined using chi-square (χ^2^) test.

## DISCUSSION

In the present study, we found that high CD82 expression suppresses development of a motile mesenchymal phenotype and rather intensifies epithelial characteristics in human prostate cancer cells adhered to the fibronectin matrix. In particular, CD82 function of inhibiting fibronectin-induced EMT appeared to be based on its inhibitory role in adhesion signaling mediated by the fibronectin-binding integrins, highlighting the importance of integrin signaling in EMT. Matrix adhesion signaling has been suggested to induce EMT, working independently or in association with other signaling pathways [36]. Matrix-dependent EMT was first found in embryonic epithelium incubated in three-dimensional gels of type I collagen, where the cells acquired a mesenchymal morphology and migratory properties [37]. Later studies revealed that type I collagen fibers directly downregulate E-cadherin and indirectly upregulate N-cadherin through the interactions with β_1_ integrins [38, 39]. Moreover, the amount of type I collagen in the matrix was increased during EMT [14], implicating a positive feedback loop between the collagen matrix and EMT. A fibronectin matrix also contributes to the development of mesenchymal phenotypes, along with providing a migration track to the cells [40]. In particular, fibronectin synthesized from EMT-derived mesenchymal cells further stimulated epithelial cells to undergo EMT [30]. Integrins activated by these matrix proteins also participate in EMT, mostly by stimulating various intracellular signaling pathways. Adhesion signaling mediated by α_2_β_1_, α_3_β_1_, α_5_β_1_, and α_v_β_3_ integrins were shown to induce EMT in association with TGF-β receptor signaling [19–24]. FAK and ILK, signaling mediators downstream of integrins, also play a role in inducing EMT [25–29], which is further demonstrated in the present study. Thus, signals derived from cell adhesion to particular matrix components induce EMT in various epithelial cell types. Therefore, epithelial cell fate could be influenced not only by changes in the composition of the surrounding matrix but also by alterations in the integrin-matrix interactions and subsequent intracellular signaling events.

Several tetraspanin proteins have been suggested to regulate the matrix-binding affinity, signaling activity, and membrane trafficking of the associated integrins in the multi-molecular TEM structure [1, 4, 35]. In various adherent cells, CD82 has been known to form physical complexes with α_3_β_1_ and α_5_β_1_ integrins and, to a lesser extent, α_6_β_1_ integrin in the TEM structure [35]. Our present study also showed a physical association of CD82 with α_3_β_1_ and α_5_β_1_ integrins in normal prostate epithelial cells. Importantly, several studies have provided evidence that CD82 inhibits integrin-mediated intracellular signaling cascades. CD82 attenuates matrix adhesion-dependent activation of Src and phosphorylation of its downstream targets such as FAK and p130Cas, leading to reduced cell adhesion and migration [41, 42]. Previously, we also found that CD82 suppresses β_1_ integrin activation by matrix adhesion and formation of a focal adhesion complex in prostate cancer cells, along with a decreased expression of fibronectin [31]. The present study also demonstrates the negative effects of CD82 on fibronectin-induced activation of Src, FAK, and ILK. Thus, CD82 represses the matrix-binding affinity and signaling activity of the integrins, resulting in reduced integrin-matrix interactions and integrin outside-in signaling.

In contrast to CD82, the tetraspanin CD151 promotes cell migration, collaborating with β_1_ integrins and PKC [43, 44]. In particular, Ke *et al.* showed that CD151 can induce EMT in hepatocellular carcinoma with the cooperation of integrin α_6_β_1_ [45]. We have also previously shown that CD151 increases motility and invasiveness of melanoma cells by stimulating β_1_ integrin-mediated signaling cascades [7, 46]. Thus, CD151 stimulates integrin signaling and acts as an EMT inducer, which is opposite to the CD82 function of suppressing both integrin signaling and EMT. Therefore, tetraspanin proteins seem likely to influence the matrix-dependent EMT program depending on how they affect integrin-mediated cellular and signaling events.

Huang *et al.* showed different degrees of EMT among ovarian carcinoma cell lines, suggesting the existence of intermediate states between epithelial and mesenchymal ends of the EMT process [47]. According to their model system, the three human prostate cancer cell lines used for gene transfection/transduction in this study also have different degrees of EMT. Notably, the EMT degrees in those parental cell lines, as well as the stable transfectant clones with different CD82 levels, appeared to be inversely correlated with CD82 expression levels, implicating that decrease or loss of CD82 expression renders prostate cancer cells prone to undergoing EMT. Furthermore, the present study defines the expression level of CD82 as a crucial factor to determine how the fibronectin matrix influences the epithelial/mesenchymal state of prostate cancer cells. Fibronectin induces EMT in prostate cancer cells with no or low levels of CD82, while it promotes MET, the reverse process of EMT, in the cells with high CD82 levels, indicating differential effects of fibronectin on epithelial-mesenchymal plasticity depending on CD82 expression levels. According to the current findings, we propose that fibronectin adhesion of prostate cancer cells with high CD82 levels does not elicit strong enough integrin signaling to upregulate E-cadherin repressor(s) such as Snail, which enables the cells to maintain an epithelial nature through continuous E-cadherin expression. However, if CD82 is downregulated below a certain level during malignant progression, fibronectin matrix can activate its receptor integrins so that prostate cancer cells receive strong enough adhesion signals to induce EMT, resulting in the development of a motile mesenchymal phenotype. This proposition is supported by the immunofluorescence staining data of human prostate cancer tissue specimens, which illustrated that CD82 was co-downregulated with E-cadherin during prostate cancer development, along with inversely upregulated mesenchymal proteins including Snail, α-SMA, and vimentin.

In conclusion, the tetraspanin membrane CD82/KAI1 is highly likely to block EMT in human prostate cells adhered to the matrix containing fibronectin by repressing integrin-mediated signaling. Our current study may not only be helpful to reveal the mechanisms underlying the invasion- and metastasis-suppressing role of CD82/KAI1, but also contribute to the comprehensive understanding of the EMT program under the control of the pericellular matrix in tumor microenvironments.

## MATERIALS AND METHODS

### Antibodies and reagents

Mouse monoclonal antibodies to N-cadherin (D-4), vimentin (V9), Snail (G-7), and the large extracellular loop of CD82 (G-2) and rabbit polyclonal antibodies to Slug (H-140), Twist (H-81), FAK (A-17), TM4SF2 (Y-19), phospho-ILK^(T173)^, and phospho-FAK^(Y925)^, and the C-terminus of CD82 (C-16) and goat polyclonal antibodies to integrin α3 (I-19), α5 (P-19), and α6 (N-19) subunits and an inhibitor to FAK (FAK inhibitor 14) were purchased from Santa Cruz Biotechnology (Santa Cruz, CA). Monoclonal Antibodies to integrin β_1_ subunit (P5D2) and α-SMA (E184) and an inhibitor to ILK (Cpd22) were purchased from Millipore (Billerica, MA). Monoclonal antibodies to E-cadherin (36/E-cadherin) and fibronectin (C6F10) were obtained from BD Biosciences (San Diego, CA). Antibodies to phospho-Src^(Y416)^ and ILK were purchased from Cell Signaling (Beverly, MA) and Abcam (Cambridge, UK), respectively. Alexa fluor-conjugated goat anti-mouse (Alexa488) and goat anti-rabbit (Alexa555) secondary antibodies were from Life Technologies (Grand Island, NY). The small interfering RNAs (siRNAs) targeting integrin α_3_, α_5_, or α_6_ subunit were obtained from Bioneer (Daejeon, Korea) and sequences are listed in the supplementary Table S2. All other reagents were from Sigma-Aldrich (St. Louis, Mo) unless indicated otherwise.

### Cell culture and generation of stable transfectant clones with increased or decreased CD82 levels

Human prostate cancer cell lines, LNCaP, DU145, and PC3 obtained from ATCC were cultured in complete medium as described previously [31]. PZ-HPV-7 (ATCC), a human prostate epithelial cell line transformed by human papillomavirus-18, was cultured in K-SFM medium (Invitrogen, Carlsbad, CA) supplemented with 0.05 mg/ml bovine pituitary extract and 5 ng/ml EGF. To generate stable CD82 transfectant clones of DU145 cells, DU145 cells were transfected with full-length CD82 cDNA subcloned into the pcDNA3 vector (Invitrogen) using Lipofectamine 2000 reagent according to the manufacturer's instructions. Stable antisense CD82 transfectant clones of LNCaP cells were generated by transfecting LNCaP cells with a reversed partial CD82 cDNA fragment (100 bases, +1 to +99) subcloned into pcDNA3. Empty pcDNA3 was transfected as a control (mock transfectant). Neomycin-resistant clones were isolated by growing the cells in complete medium containing 0.4 mg/ml G418 (Invitrogen). CD82 expression levels in stable transfectant clones were examined by RT-PCR and immunoblotting analyses.

### Preparation of recombinant adenovirus and cell infection

Recombinant adenoviruses, expressing the wild-type or LEL mutant of CD82, were generated using the AdEasy™ adenoviral vector system (Agilent Technologies, Santa Clara, CA). Briefly, CD82 cDNA was first cloned into the pShuttle vector. *E. coli* BJ5183 cells were co-transformed with the linearized pShuttle vector and pAdEasy-1 adenoviral vector. Q293A adenovirus packaging cells were then transfected with the resultant recombinant Ad plasmid using Lipofectamine reagent. Primary virus stocks prepared by cell freeze-thaw and subsequent centrifugation were amplified in Q293A cells and subsequently purified by ultracentrifugation in two discontinuous CsCl_2_ gradients, followed by ultrafiltration. For viral infection, titrated viral stocks were suitably diluted in complete medium to obtain the desired multiplicity of infection (MOI) and added to cell monolayers. After a 24 hour incubation, the virus-containing medium was replaced by fresh medium for further analysis.

### Cell motility assay

Chemostatic cell movement was analyzed using an Oris™ cell migration assay kit (Platypus Technologies, Madison, WI) as described previously [48]. For the chemotactic cell migration assay, a Transwell-chamber insert (Corning Costar, Cambridge, MA) with filters precoated on the lower surface with gelatin was placed in a 24-well tissue culture plate containing conditioned medium from NIH3T3 fibroblasts cultured in complete medium. Cells (2 × 10^4^) suspended in serum-free RPMI medium were added to the upper chambers. Following a 24 hour incubation, cells were fixed and stained with crystal violet. After removal of non-migrated cells on the upper surface of the filter, chemotactic cell motility was quantified by counting the cells on the lower surface of the filter.

### Invasion assay

An *in vitro* invasion assay into matrigel was performed as described previously [46]. Additionally, an *in vivo* cancer cell invasion assay was conducted using 11-day-old chick embryos, wherein cells (1 × 10^5^), labeled with a fluorescent probe for long-term tracing of living cells, CMTMR (Invitrogen), were suspended in serum-free DMEM (100 μl), and seeded atop the chick chorioallantoic membrane (CAM) as described previously [49]. After a three day incubation in a humidified stationary incubator at 38°C, the embryos were snap-frozen in liquid nitrogen and cross-sectioned with a microtome. Following staining with DAPI, 20 μm-thick CAM cryosections were viewed under a fluorescent microscope (Olympus).

### Human prostate cancer tissue microarray and immunofluorescence stanining

Two commercial human prostate cancer tissue microarrays (AccuMax™ TMAs) were obtained from Petagen Incorporation (Seoul, Korea): TMA A222, lot 122120312171, contained malignant samples in duplicates from 45 patients of which 4 were also used to provide single-spot non-neoplastic control tissue. TMA A223(II), lot 122120609121, contained 45 prostate cancer patient samples in duplicates and provided 8 single control spots. The TMA's accompanying pathological data are described in the Supplementary Table S3. For immunofluorescence stanining, microarray slides containing consecutive sections of human prostate tumors were deparaffinized and autoclaved for 15 min in citrate buffer (pH 6.0). After blocking with bovine serum albumin, the slides were incubated with rabbit anti-CD82 antibody, together with mouse anti-E-cadherin or anti-vimentin antibody for 3 hours at room temperature. After washing with PBS, the sections were incubated with AlexaFluor555 (red)-conjugated anti-rabbit IgG and AlexaFluor488 (green)-conjugated anti-mouse IgG for 2 hours. Following DAPI staining, fluorescence images of the tissues were taken under a fluorescent microscope.

### Other analysis/assays

Immunoprecipitation and immunoblotting analyses, and siRNA transfection were performed as described previously [7, 46, 48].

### Statistical analysis

Different numbers between two groups were analyzed by the student's *t*-test. Immunohistochemistry analysis was performed using Pearson's χ^2^ test. A value of *p* < 0.03 was considered statistically significant. Correlation between the immunofluorescence intensity scores of WCD82 and E-cadherin or vimentin was determined using the Spearman and Pearson correlation coefficient tests.

## SUPPLEMENTARY FIGURES AND TABLES




