# Supplementary material for: The metastasis suppressor CD82/KAI1 inhibits fibronectin adhesion-induced epithelial-to-mesenchymal transition in prostate cancer cells by repressing the associated integrin signaling
Source: Oncotarget. 2016 Dec 1;8(1):1641–54. doi: 10.18632/oncotarget.13767 (PMC5352085; doi:10.18632/oncotarget.13767)
Supplement: Supplementary file 1 [file oncotarget-08-1641-s001.pdf]

The metastasis suppressor CD82/KAI1 inhibits fibronectin adhesion-induced epithelial-to-mesenchymal transition in prostate cancer cells by repressing the associated integrin signaling

SUPPLEMENTARY FIGURES AND TABLES

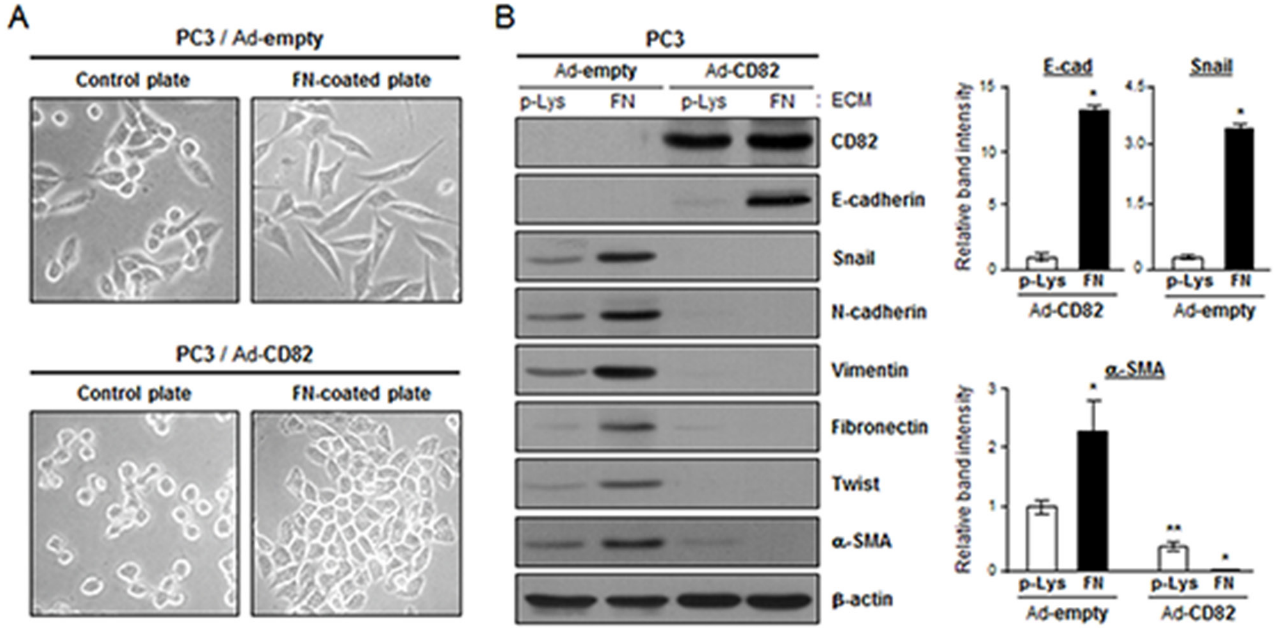

Supplementary Figure S1: Inhibition of fibronectin-induced EMT by ectopically expressed CD82 in PC3 cells. Following infection with adenovirus containing a CD82 expression construct, PC3 cells grown on uncoated plates (control) or poly-L(+)-lysine (p-Lys)- or fibronectin (FN)-coated plates were examined for cell morphology **A.** and expression of E-cadherin and mesenchymal proteins **B.** *$p < 0.01$ *versus* p-Lys; **$p < 0.03$ *versus* Ad-empty.

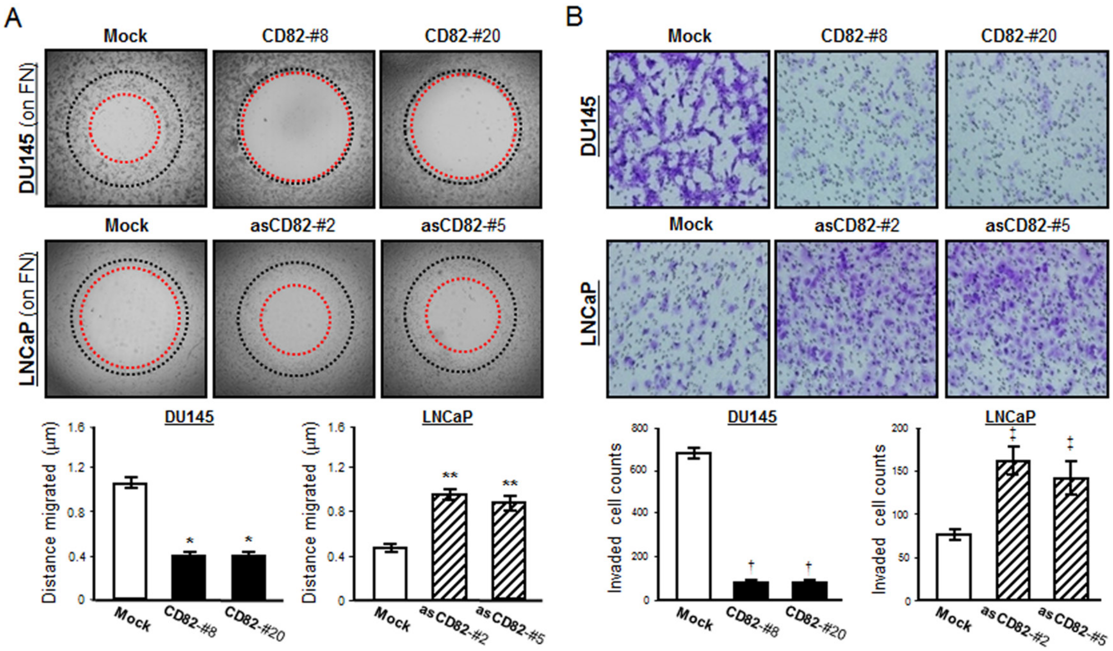

Supplementary Figure S2: CD82 inhibition of chemostatic motility and invasiveness of prostate cancer cells. Cell migration into a wound area **A.** and invasion into matrigel **B.** were measured by using an Oris™ cell migration assay kit and a Transwell-chamber invasion assay system, respectively. *, **, and ‡, $p < 0.03$ *versus* mock; †, $p < 0.01$

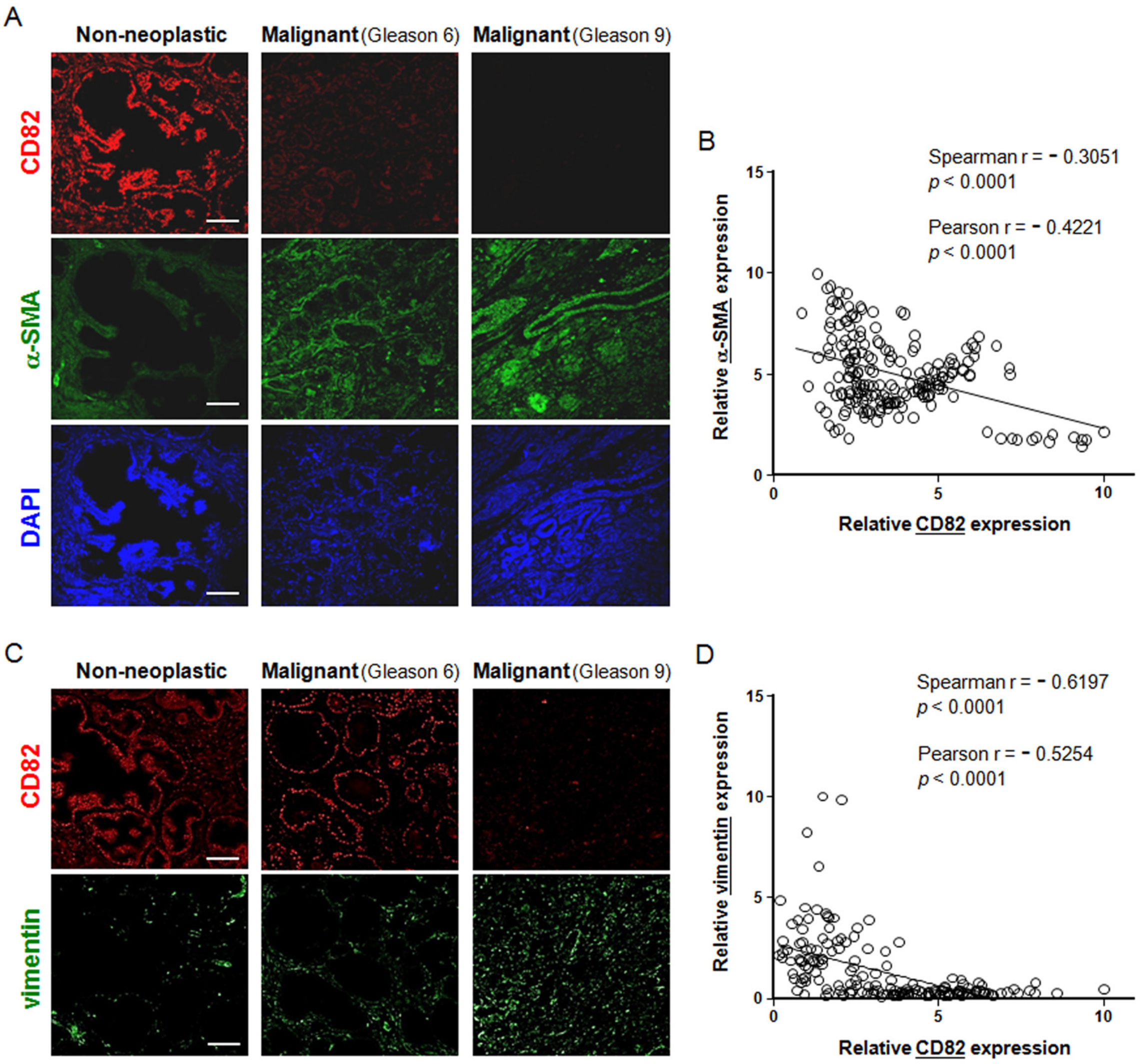

Supplementary Figure S3: Expression levels of CD82, α-smooth muscle actin, and vimentin in human prostate cancer tissue samples. A, C. Two microarray slides, which contain 90 human prostate cancer tissue specimens and 12 normal human prostate tissue samples, were subjected to immunofluorescence staining using rabbit anti-CD82 antibody, together with mouse antibodies to α-smooth muscle actin (α-SMA) (A) or vimentin (C). Scale bar, 100 μm. **B, D.** Relationship of CD82 expression levels with α-SMA (B) or vimentin (D) levels was examined by assessing fluorescence intensity of target proteins in each tissue specimen.

Supplementary Table S1: Expression of CD82, α-SMA, and vimentin in human prostate cancer tissue samples

Relative expression of target protein[a]	Percentage of cases of histologic type (n, total number)[b]			
		Malignant		
	Non-neoplasitic (n=12)	≦ Gleason 7 (n=66)	Gleason 8 ≦ (n=101)	*p* value[c]
**CD82**				
1	0% (0)	15.2% (10)	50.5% (51)	< 0.0001
2	0% (0)	77.3% (51)	43.6% (44)	
3	25.0% (3)	7.6% (5)	5.9% (6)	
4	75.0% (9)	0% (0)	0% (0)	
**α-SMA**				
1	100% (12)	4.5% (3)	0% (0)	< 0.0001
2	0% (0)	69.7% (46)	30.7% (31)	
3	0% (0)	25.8% (17)	44.6% (45)	
4	0% (0)	0% (0)	24.8% (25)	
**vimentin**				
1	100% (12)	90.9% (60)	75.2% (76)	< 0.0001
2	0% (0)	9.1% (6)	18.8% (19)	
3	0% (0)	0% (0)	3.0% (3)	
4	0% (0)	0% (0)	3.0% (3)	

[a]Staining intensity relative to the highest intensity was scored as follows: 1, < 25%; 2, 25~50%; 3, 50~75%; 4, >75%
[b]Proportion of tumors with respective score out of total tissue number (*n*) examined is given.
[c]Statistical significance was determined using chi-square (χ^2) test.

Supplementary Table S2: siRNA sequences (sense)

Integrin α_3-specific siRNA combination
5’-GCGCAAGGAGUGGGACUUAdTdT-3’
5’-UUACAGAGACUUUGACCGAdTdT-3’
Integrin α_5-specific siRNA combination
5’-UCACAUCGCUCUCAACUUCdTdT-3’
5’-GAACGAGUCAGAAUUUCGAdTdT-3’
Integrin α_6-specific siRNA combination
5’-CCAUCACAGUAACUCCUAAdTdT-3’
5’-GGAUAUGCCUCCAGGUUAAdTdT- 3’

Supplementary Table S3: The clinopathological data of the tumor specimens on the human prosate cancer tissue microarray

See Supplementary File 1
